# Image fraud in nuclear medicine research

**DOI:** 10.1007/s00259-025-07515-5

**Published:** 2025-08-16

**Authors:** Robert M. Kwee, Andreea M. Pavel, Thomas C. Kwee

**Affiliations:** 1 Department of Radiology, Zuyderland Medical Center, Heerlen/Sittard/Geleen, The Netherlands; 2 Medical Imaging Center, Departments of Radiology, Nuclear Medicine, and Molecular Imaging, University of Groningen, University Medical Center Groningen, Groningen, The Netherlands

**Keywords:** Fraud, Nuclear medicine, Research, Scientific misconduct

## Abstract

**Purpose:**

To assess nuclear medicine researchers’ experiences and attitudes toward image fraud, as well as their perspectives on preventive measures.

**Methods:**

This survey targeted corresponding authors who published in three nuclear medicine journals between 2021 and 2024. Participants were asked about their experiences related to medical image fraud, as well as their views on its prevalence, causes, and potential preventive measures.

**Results:**

Of the 2,837 corresponding authors invited, 284 (10.0%) completed the survey. Most of the 284 respondents were mid-career European male MDs with over 10 years of research experience. While 91% reported never feeling pressured to falsify medical images, 13.7% admitted doing so in the past five years, and 38.7% had witnessed colleagues engaging in such practices. Common forms included cherry-picking, unauthorized image reuse, and misleading enhancements. In the past five years, 1.1% admitted using AI to falsify medical images, while 2.8% reported witnessing colleagues do so. No demographic factors were significantly associated with misconduct. Key drivers cited were publication pressure, competition, and aesthetic expectations. Respondents emphasized the need for greater transparency, oversight, and cultural change. Current safeguards were generally considered ineffective. Stricter policies, increased awareness, and AI tools were suggested as potential solutions.

**Conclusions:**

Image fraud in nuclear medicine research appears to be relatively prevalent. It is more frequently witnessed among other colleagues than self-reported by individual researchers. The findings highlight the need to fostering a culture of research integrity and for stronger preventive measures, including greater awareness, stricter journal policies, and improved control.

**Supplementary Information:**

The online version contains supplementary material available at 10.1007/s00259-025-07515-5.

## Introduction

Scientific integrity is the cornerstone of credible research and medical advancement. In the field of nuclear medicine, where visual data often serve as key evidence in publications about both diagnostic and therapeutic innovations, the accuracy and authenticity of published images are critical. Unfortunately, image fraud exists. A previous investigation found that 3.8% of biomedical research articles published between 1995 and 2014 contained duplicated images, with at least half showing signs of intentional manipulation [1]. These unethical practices undermine the validity of individual studies, may lead to misinformed clinical decisions, and erode public trust in scientific credibility. The rapid development of artificial intelligence (AI) has become a new concern regarding image fraud in scientific publications. Current AI technology can generate fake images, plagiarize existing images, and modify images in ways that are extremely difficult to detect through visual inspection alone [2–4]. The highly specialized and visual nature of nuclear medicine imaging - such as PET, SPECT, and hybrid modalities - makes the field particularly susceptible to image fraud. In addition, the increasing pressure to publish in high-impact journals and the reliance on imaging results for statistical significance may further incentivize misconduct [5]. Little is known about how researchers in the nuclear medicine community perceive image fraud, how frequently they encounter it, and what mechanisms they believe are effective in preventing it. Understanding these perspectives is essential for developing targeted interventions, improving editorial policies, and fostering a culture of research integrity. To address this, we surveyed researchers to assess their experiences and attitudes toward image fraud, as well as their views on potential countermeasures.

## Methods

### Study design

This prospective survey study received approval from the Institutional Review Board of the University Medical Center Groningen. Eligible participants included all corresponding authors who published articles between 2021 and 2024 in one of the top three general nuclear medicine journals, as ranked by the 2023 Journal Citation Reports [6]: *Journal of Nuclear Medicine*, *Clinical Nuclear Medicine*, or the *European Journal of Nuclear Medicine and Molecular Imaging*. Corresponding authors were invited by email to complete a survey addressing medical image fraud in scientific literature. The survey was administered online through a web link generated using specialized software (Qualtrics, Provo, UT). To enhance response rates, 5 weekly reminder emails were sent. Participation was entirely voluntary and anonymous. Corresponding authors were excluded if they lacked a valid email address, had an automatic out-of-office reply indicating an extended absence, or were personally known to the study authors. The software was set up to ensure each respondent could complete the survey only once.

### Survey

Participants were initially asked to provide demographic and professional information, including age, gender, country of employment, academic degree, academic position, and years of research experience (based on their own interpretation of the term “research experience”). They were then questioned about whether, within the past five years, they had felt pressure from colleagues, supervisors, or reviewers to alter medical images in a way that could misrepresent data. Next, participants were asked whether they had personally engaged in any of the following practices over the past five years: enhancing images in a way that misrepresents data or findings; adding or removing features (e.g., tissue or pathology) in medical images; duplicating or reusing images without formal permission; fabricating medical images; cherry-picking images to support specific conclusions; or using AI to falsify medical images for publication. They were also asked if they had witnessed any of these behaviors among colleagues during the same period. Additionally, participants were asked whether they believe image fraud is common in their field; to identify the main reasons researchers may falsify medical images in scientific publications; and to assess the effectiveness of current journal policies, peer-review processes, and institutional guidelines in preventing or detecting such misconduct. Participants were allowed to select multiple responses for certain survey questions, as indicated by instructions in the questionnaire. They were also invited to suggest measures to reduce image fraud in scientific research. An open comment section was provided at the end of the survey. The full survey is presented in Supplementary File 1. The survey was not formally validated, with questions and answer options developed by the authors based on relevant literature, prior experience, and internal consensus.

### Data analysis

Participants’ characteristics and responses to questions regarding medical image falsification in the scientific literature were analyzed descriptively. Logistic regression analyses were conducted to assess associations between participant characteristics (age, gender, continent of work, academic degree, academic position, and years of research experience) and engagement in image fraud. The latter was defined as any of the following actions: enhancing images in a way that misrepresents data or findings, adding or removing features in medical images, duplicating or reusing images without formal permission, fabricating medical images, or cherry-picking images to support specific conclusions. Using AI to falsify medical images for publication was also considered image fraud. A *P*-value of less than 0.05 was considered statistically significant. All statistical analyses were conducted using IBM SPSS Statistics, version 28. Participant comments submitted at the end of the survey were analyzed qualitatively to identify recurring themes.

## Results

### Participants

Of the 2837 corresponding authors invited and with valid email addresses, 284 (10.0%) completed the survey. The majority of respondents were aged 35–44 years (30.3%), male (68.7%), based in Europe (62.0%), held a medical doctor (MD) degree (64.4%), were full professor (31.3%), and had over 10 years of research experience (76.8%) (Table 1).

**Table 1:** Characteristics of 284 participating corresponding authors

Variable	Category	No. (%)
Age distribution	25–34 years	37 (13.0%)
	35–44 years	86 (30.3%)
	45–54 years	75 (26.4%)
	55–64 years	49 (17.3%)
	> 65 years	37 (13.0%)
Gender	Male	195 (68.7%)
	Female	88 (30.9%)
	Other	1 (0.4%)
Continent	Europe	176 (62.0%)
	North America	57 (20.1%)
	Asia	44 (15.5%)
	Australia	4 (1.4%)
	South America	3 (1.0%)
Academic degree	Medical degree, with or without other degree	183 (64.4%)
	Other degree	101 (35.6%)
Academic position	Full professor	89 (31.3%)
	Associate professor	56 (19.7%)
	Assistant professor	36 (12.7%)
	Fellow or resident	25 (8.8%)
	Instructor/lecturer	15 (5.3%)
	Other/none	63 (22.2%)
Research experience	< 5 years	17 (6.0%)
	5–10 years	49 (17.2%)
	> 10 years	218 (76.8%)

### Frequency of medical image falsification

A total of 258 participants (90.8%) reported never feeling pressured by colleagues, supervisors, or reviewers in the past five years to alter medical images in a way that could misrepresent data. In contrast, 22 participants (7.7%) occasionally felt such pressure, and 4 (1.4%) reported feeling pressured frequently. Thirty-nine participants (13.7%) admitted to having engaged in some form of medical image falsification within the past five years, while 110 (38.7%) reported witnessing such practices by colleagues during the same period. The most commonly reported form of image falsification was the selective presentation of nonrepresentative images to support conclusions (“cherry picking”) (48.3%), followed by duplicating or reusing images without proper authorization (20.9%), and enhancing images in ways that misrepresented the underlying data or findings (19.1%) (Table 2). Three participants (1.1%) disclosed using AI to falsify medical images for publication in the past five years, and eight (2.8%) reported witnessing colleagues do the same. When asked about the perceived prevalence of medical image falsification in scientific publications, 37 participants (13.0%) considered it extremely rare or nonexistent, 146 (51.4%) described it as rare, 47 (16.5%) as somewhat common, and 5 (1.8%) as very common, while 49 participants (17.3%) were uncertain.

**Table 2:** Reported medical image falsification by survey respondents and witnessed medical image falsification among colleagues in the past five years

Type of medical image falsification	Survey respondents	Colleagues	Total No. (%)
Cherry-picking images to support conclusions (i.e. selectively choosing specific, nonrepresentative images that confirm a desired result or argument)	29	82	111 (48.3%)
Duplicating or reusing images without formal permission	6	42	48 (20.9%)
Enhancing images in such a way that it results in the misrepresentation of data or findings	5	39	44 (19.1%)
Removing or adding features (e.g. tissues or pathology) in medical images	6	17	23 (10.0%)
Fabricating medical images	2	2	4 (1.7%)

### Determinants of medical image falsification

There were no significant associations between participants’ age, gender, continent of work, academic degree, academic position, or years of research experience and self-reported instances of medical image falsification in scientific literature over the past five years (Table 3).

**Table 3:** Multivariate analysis of the association between participants’ characteristics and medical image falsification in the scientific literature over the past five years

Variable	Category	Odds ratio	95% CI	*P*-value
Age	NA	0.669	0.426 to 1.049	0.080
Gender^1^	Female	0.643	0.284 to 1.454	0.289
Continent of work^2^	North America	0.341	0.093 to 1.254	0.105
	Asia	1.616	0.663 to 3.936	0.291
Academic degree^3^	Other degree than medical doctor	1.295	0.607 to 2.763	0.504
Academic position^4^	Associate professor	1.326	0.413 to 4.254	0.636
	Assistant professor	1.935	0.524 to 7.141	0.322
	Fellow or resident	2.301	0.479 to 11.052	0.298
	Instructor/lecturer	2.722	0.629 to 11.781	0.180
	Other/none	1.427	0.435 to 4.686	0.557
Years of research experience	NA	1.407	0.628 to 3.149	0.406

Abbreviations: *CI*: confidence interval, *NA*: not applicable

^1^Male gender was used as reference category

^2^Europe was used as reference category

^3^Medical doctor degree was used as reference category

^4^Full professor was used as reference category

### Causes and potential solutions of medical image falsification

A total of 282 participants provided insights into why researchers might falsify medical images in scientific literature. The three most commonly cited reasons were pressure to publish in high-impact journals (30.3%), competition for funding and academic positions (23.4%), and the expectation to generate visually appealing images (22.5%) (Table 4). Regarding the effectiveness of current safeguards, 45 participants (15.8%) viewed journal policies, peer-review processes, and institutional guidelines as not effective at all in preventing or detecting image falsification. Another 120 (42.3%) considered them not very effective, 77 (27.1%) rated them as somewhat effective, and only 9 (3.2%) found them highly effective, while 33 participants (11.6%) were unsure. Additionally, 283 participants suggested strategies to reduce medical image falsification. The most frequently proposed measures were stricter journal policies and image integrity checks (22.6%), greater awareness and discussion of the issue (20.0%), and the adoption of AI-based tools to detect manipulated images (19.7%) (Table 5).

**Table 4:** Reasons for medical image falsification in scientific publications according to study participants (*n* = 282)

Reason	No. (%)
Pressure to publish in high-impact journals	214 (30.3%)
Competition for funding and academic positions	165 (23.3%)
Expectation to produce visually appealing images	159 (22.5%)
Unawareness of ethical boundaries in image processing	82 (11.6%)
Lack of strict enforcement of ethical guidelines	79 (11.2%)
Other^1^	8 (1.1%)

^1^Quoted reasons (responses are quoted verbatim, including any spelling or grammatical errors, to preserve their original meaning and to avoid misinterpretation or misrepresentation): “Not always aware that alteration may lead to misinterpretation (because they misinterpret themselves sometimes as well)”, “Lack of quantification and incompetence”, “Images, including graphical abstracts and hypothesis supporting schematic graphics, have almost become an (overrated and potentially misleading) necessity for review and eventual acceptance in many, even highly ranked journals”, “Pressure from mentors/suprvisors in case of students/postdocs/juniors”, “Often lack of clear case examples”, “Natural, unconscious perception bias (tendency to select confirming evidence/blindness to disconfirming evidence)”, “The proximal reason would probably because they have rationalized that it is acceptable to do so. The impetus for their rationale could be many possibilities, and I don’t see any reason to speculate.”, “Reviewers of manuscripts and grant applications. The problem is if you show one incongruent data point then everyone focuses on that and forgets about 999 congruent data points and ask unnecessary questions and experiments to show that deviation was irrelevant.”

**Table 5:** Measures that could be implemented to reduce medical image falsification in scientific research according to study participants (*n* = 283)

Measure	No. (%)
Stricter journal policies and image integrity checks	153 (22.6%)
Increased awareness and discussion about the issue	135 (20.0%)
Use of AI-based tools to detect manipulated images	133 (19.7%)
Stronger consequences for researchers found guilty of falsification	127 (18.8%)
Better training on ethical image processing	123 (18.2%)
Other^1, 2^	5 (0.7%)

Abbreviations: *AI*: artificial intelligence

^1^ Quoted suggested measures (Responses are quoted verbatim, including any spelling or grammatical errors, to preserve their original meaning and to avoid misinterpretation or misrepresentation): “Transparent peer review (with raw data)”, “Requesting reviewers to focus on the issue”, “ask for the"brute"original images”, “Tools to systematically screen for falsified images. If you are not looking for it, people will submit them without fear of being caught.”, “As stated above. If reviewers, editors and granting agencies can start accepting that randomness is part of experimental observation that will help the community.”

^2^The following comment was not included in the count but is provided here for completeness: “My concern about AI is who is checking to determine that the AI is correct? I have personally observed that AI is perfectly willing to report verifiable falsehoods.”

### Principal themes emerging from participant responses (Supplementary file 2)

- Stronger reporting and oversight needed

- Participants highlighted the importance of requiring detailed documentation of image acquisition protocols, reconstruction methods, and processing steps. They called for stricter journal standards and more rigorous editorial and peer-review oversight to ensure transparency and reproducibility.

- Systemic and cultural pressures drive questionable practices

- Many noted that pressures to publish in high-impact journals, competition for funding, and meeting industry expectations contribute to practices such as cherry-picking data, selective image enhancement, and image misrepresentation.

- Diverse views on prevalence and solutions

- While some believed image falsification is rare (especially in high-impact journals), others expressed concern about its potential under-detection. Participants suggested increased education, awareness, and open discussion, with mixed opinions on the reliability and role of AI-based detection tools.

## Discussion

This study provides one of the first insights into how researchers in the field of nuclear medicine perceive, experience, and respond to medical image fraud in scientific publications. Most respondents (90.8%) reported never feeling pressured to manipulate images, and relatively few declared to engaging in misconduct themselves. However, the fact that over one in three participants had witnessed such practices among colleagues may suggest a concerning level of underreported or tacitly accepted behavior within the field. Cherry-picking of images emerged as the most common form of image manipulation, highlighting a gray area where subjective image selection can blur into intentional misrepresentation. It can be considered a form of selective reporting, which the general public overwhelmingly views as immoral and deserving of punishment, according to a study by Pickett et al. [7]. It may be recognized that researchers rarely select images at random, and that readers may not expect this. However, labeling an image as “typical” implies representativeness, which is problematic without a clear selection rationale. Current norms and incentives push researchers toward such practices, even unintentionally, and risk biasing interpretation. While requiring full image databases may be impractical, greater transparency is both feasible and necessary. Simple measures, such as stating how images were selected or providing representative sets as supplementary material, could help. Overall, our findings underscore the need for greater transparency in image reporting, stronger institutional and editorial safeguards, and a cultural shift that focuses less on individual blame and more on addressing the systemic pressures that contribute to questionable research practices.

A particularly striking observation from this study is the limited perceived effectiveness of current mechanisms designed to prevent image fraud. Journal policies, peer review, and institutional guidelines - cornerstones of research oversight - were generally rated as inadequate by respondents. This perception may reflect inconsistencies in enforcement, lack of technical training among reviewers, or editorial constraints that prioritize novelty and visual appeal over methodological rigor. These findings suggest a critical need to standardize image submission protocols and to implement pre-publication integrity checks, such as automated detection tools and raw data verification.

The role of AI in image manipulation presents a dual-edged challenge. While only a very small proportion of respondents admitted to (1.1%) or had witnessed (2.8%) the use of AI for image falsification, the (rising) potential for such tools to be misused is considerable. AI technologies can generate hyper-realistic, falsified medical images that evade human scrutiny [2–4]. Simultaneously, AI offers promising avenues for detection, with emerging algorithms capable of flagging image anomalies or suspicious patterns. The mixed attitudes expressed by participants reflect both optimism and uncertainty about AI’s reliability and its potential integration into editorial workflows. Importantly, any AI-based detection strategy must be paired with human oversight and ethical guidelines to avoid false accusations and ensure fair evaluation.

Cultural and structural factors also play a central role in shaping image-related misconduct. Many respondents cited the pressure to publish in high-impact journals, secure funding, and produce visually compelling data as key motivators behind unethical practices. These drivers are not unique to nuclear medicine but are perhaps magnified in a discipline where images often serve as primary endpoints. Reforming this culture will require systemic changes: realigning academic incentives, promoting open science practices, and encouraging mentorship that prioritizes integrity over productivity. Initiatives such as registered reports, mandatory image provenance declarations, and open peer review may help recalibrate expectations and reduce perverse incentives.

Our findings align with previous surveys across medical imaging disciplines, which report that scientific fraud, publication bias, and honorary authorship are relatively common despite generally high confidence in research integrity [8–12]. For example, previous studies reported that 4–6% of radiology, cardiovascular imaging, neuroradiology, and nuclear medicine researchers admitted to fraud, with 20–30% suspecting misconduct in their departments [8–12]. Publication bias and undeserved authorship were also frequently reported. Factors associated with fraud include junior academic rank and working in countries with higher corruption indices [9]. The results of the present study builds on these previous works by focusing specifically on image fraud in nuclear medicine, highlighting emerging challenges related to digital manipulation and AI tools not fully addressed in prior work.

Our findings highlight a threat to research integrity in nuclear medicine. They also point toward actionable pathways for reform. Greater awareness, community dialogue, and policy innovation are essential to foster an environment in which transparency is rewarded and misconduct is effectively deterred. Future research should explore the feasibility and effectiveness of specific interventions, such as mandatory image audits, AI-assisted peer review, and ethics training tailored to imaging researchers. Engaging editors, reviewers, and institutional leaders in this work will be key to designing practical, field-wide solutions.

Our study has some limitations. First, the data rely on self-reported responses, which may be subject to recall bias or social desirability bias, potentially leading to underreporting of misconduct. Second, the survey targeted corresponding authors of publications in high-impact nuclear medicine journals, which may not reflect the experiences of researchers in lower-tier journals. Third, while the response rate was reasonable, non-participants may differ from participants, introducing potential selection bias. Fourth, the survey question on image duplication focused on the lack of formal permission, which primarily relates to copyright or intellectual property issues. However, the more ethically significant concern (namely, whether duplicated or re-used images were clearly labeled or disclosed in the publication) was not directly addressed, and this distinction may have influenced how respondents interpreted the question. Fifth, invitees were asked only about external pressures to manipulate images, while internal factors such as personal ambition or vanity that may also strongly contribute to such behavior were not explored.

In conclusion, image fraud in nuclear medicine research appears to be relatively prevalent. It is more frequently witnessed among other colleagues than self-reported by individual researchers. The findings highlight the need for stronger preventive measures, including greater awareness, stricter journal policies, and improved control. Addressing systemic pressures and fostering a culture of research integrity are essential to reducing image-related misconduct.

## Supplementary Information

Below is the link to the electronic supplementary material.

ESM 1 (DOCX 24.2 KB)

ESM 2 (DOCX 25.0 KB)

## Data Availability

The datasets generated during and/or analysed during the current study are available from the corresponding author on reasonable request.
