# Supplementary material for: Image fraud in nuclear medicine research
Source: Eur J Nucl Med Mol Imaging. 2025 Aug 16;53(2):1348–53. doi: 10.1007/s00259-025-07515-5 (PMC12830451; doi:10.1007/s00259-025-07515-5)
Supplement: Supplementary file 1 — (DOCX 24.2 KB) [file 259_2025_7515_MOESM1_ESM.docx]

**Supplementary file 1.** Survey on medical image falsification in the scientific literature.

Q1 How old are you?

- Under 18
- 18-24 years old
- 25-34 years old
- 35-44 years old
- 45-54 years old
- 55-64 years old
- 65+ years old

Q2 What is your gender?

- Male
- Female
- Other

Q3 In which country do you work?

- Argentina
- Australia
- Austria
- Belgium
- Brazil
- Canada
- China
- Denmark
- Egypt
- Finland
- France
- Germany
- Greece
- India
- Ireland
- Italy
- Japan
- Korea
- New Zealand
- Norway
- Poland
- Portugal
- Saudi Arabia
- Spain
- Sweden
- Switzerland
- The Netherlands
- Turkey
- United Kingdom
- United States of America
- Other, namely: __________________________________________________

Q4 What is your academic degree? <div>(multiple options possible)</div>

- Medical doctor (MD)
- Doctor of philosophy (PhD)
- Master of science (MSc)
- Bachelor of science (BS)
- Master of public health (MPH)
- Other (please describe in box below) __________________________________________________

Q5 Which academic position do you hold?

- None
- Fellow/resident
- Instructor/Lecturer
- Assistant professor
- Associate professor
- Full professor
- Other (please describe in box below) __________________________________________________

Q6 How many years of research experience do you have?

- <5 years
- 5 to 10 years
- >10 years

Q7 Have you felt pressured by colleagues, supervisors, or reviewers in the past 5 years to alter medical images in a way that could misrepresent the data? <div></div>

- Yes, frequently
- Yes, occasionally
- No, never

Q8 Which of the following practices have you personally engaged in in published research in the past 5 years? <div> <div> <div> (please select all options that apply)</div> </div> </div>

- Enhancing images in such a way that it results in the misrepresentation of data or findings
- Removing or adding features (e.g. tissues or pathology) in medical images
- Duplicating or reusing images without formal permission
- Fabricating medical images
- Cherry-picking images to support conclusions (i.e. selectively choosing specific, nonrepresentative images that confirm a desired result or argument)
- ⊗None of the above

Q9 Have you used AI technology to falsify medical images for publication in the past 5 years?<div> <div> <div></div> </div> </div>

- Yes
- No

Q10 Which of the following practices have you witnessed <u><strong>among colleagues</strong></u> in published research in the past 5 years?<br> (please select all options that apply) <div> <div> <div></div> </div> </div>

- Enhancing images in such a way that it results in the misrepresentation of data or findings
- Removing or adding features (e.g. tissues or pathology) in medical images
- Duplicating or reusing images without formal permission
- Fabricating medical images
- Cherry-picking images to support conclusions (i.e. selectively choosing specific, nonrepresentative images that confirm a desired result or argument)
- ⊗None of the above

Q11 Have you witnessed <u><strong>colleagues </strong></u>using AI technology to falsify medical images for publication in the past 5 years?<div> <div> <div></div> </div> </div>

- Yes
- No

Q12 How common do you think medical image falsification is in scientific publications within your field? <div> <div> <div></div> </div> </div>

- Very common
- Somewhat common
- Rare
- Extremely rare or nonexistent
- Unsure

Q13 What do you think are the main reasons researchers may falsify medical images in scientific publications?<br> (please select all options that apply)<br><div> <div> <div></div> </div> </div>

- Pressure to publish in high-impact journals
- Competition for funding and academic positions
- Lack of strict enforcement of ethical guidelines
- Expectation to produce visually appealing images
- Unawareness of ethical boundaries in image processing
- Other (please specify) __________________________________________________

Q14 Do you believe current journal policies, peer-review processes, and institutional guidelines are effective in preventing and/or detecting medical image falsification? <div> <div> <div></div> </div> </div>

- Yes, highly effective
- Somewhat effective
- Not very effective
- Not effective at all
- Unsure

Q15 Which measures do you think could be implemented to reduce medical image falsification in scientific research?<br> (please select all options that apply)<br>

- Stricter journal policies and image integrity checks
- Better training on ethical image processing
- Stronger consequences for researchers found guilty of falsification
- Increased awareness and discussion about the issue
- Use of AI-based tools to detect manipulated images
- Other (please specify) __________________________________________________

Q16 Please feel free to add any narrative comments:

________________________________________________________________

________________________________________________________________

________________________________________________________________

________________________________________________________________

________________________________________________________________

End of Block: Question Tour Block 1
