# Supplementary material for: Image fraud in nuclear medicine research
Source: Eur J Nucl Med Mol Imaging. 2025 Aug 16;53(2):1348–53. doi: 10.1007/s00259-025-07515-5 (PMC12830451; doi:10.1007/s00259-025-07515-5)
Supplement: Supplementary file 2 — (DOCX 25.0 KB) [file 259_2025_7515_MOESM2_ESM.docx]

**Supplementary file 2**. Narrative responses from 31 participants provided at the end of the survey (responses are quoted verbatim, including any spelling or grammatical errors, to preserve their original meaning and to avoid misinterpretation or misrepresentation).

| **No.** | **Comment** |
| --- | --- |
| 1 | When submitting the manuscript, request the author to include a detailed description of the image acquisition protocols and reconstruction method. Or request the author to submit a checklist include detailed image processing. With consideration for personal information, request the author to upload raw data before image processing and check whether the images can be reproduced correctly. |
| 2 | This is an important topic although I have not experienced it personally. I am working in the field of MRI and PET imaging so that I can not say much about microscopic images etc. |
| 3 | There are shades of gray here. If you have an imaging test to identify disease that is 80% sensitive, it is common to show a "clear true positive: case and one also should show a "false negative case" or a false positive case. So you select a very clearly positive image for your true positive. You may narrow the gray scale our use color to enhance a finding. The finding is there but you can window in different ways. I consider this appropriate, but one can "enhance" too much and end up with potentially dubious claims of success. So, images are a digital continuum of data and the right scaling should be applied, but what is "right" depends. For example, on a CT scan, there are bone, lung, brain, liver , soft tissue windows. All look different but are showing the same data, but in a bone window you don't see the lung and in a lung window the bone looks all the same (white). So, this is rational and appropriate image enhancement to show a finding. The underlying data are not changed. So, don't confuse windowing to show a finding wiht "data manipulation" of deceptive intent. |
| 4 | The way we use publications to support careers rather than patients is just simply wrong |
| 5 | The scientific peer-review process is a trust-based system. Trust is mutually beneficial. Few journals have the resources to "enforce" anything. The few researchers and institutions who violate this trust are ostracized. A more "rigorous" system with checks and penalties is neither affordable nor collegial and likely undesirable. For example, marriage proposal is a trust based system. Instituting checks and penalties to this system probably degrades rather than improves the system. |
| 6 | Thanks for the survey which has, by itself, raised my awareness of this issue. |
| 7 | thank you |
| 8 | Nothing to add, appropriate survey. |
| 9 | "Many medical researchers do research for personal prestige only, i.e. impact factors and citation indices are valued much more than the scientific content. Without a change of culture, image falsification will always be driven by the desire to get more prestigious result. Also, clinicans often just want 'nice images', no matter what the content ist. Mathematics, high-energy physics and astrophysics managed to avoid such a culture. " |
| 10 | It is important to teach the relevant ethics and to ensure that all relevant and valid results are publishable. Often researchers struggle for yeas to obtain publishable results when negative results are known not to be acceptable to many journals. |
| 11 | "In the following, I like to comment on common mispractices regarding the reporting of (preclinical) imaging results of novel radiotracers. The most common way of ""image falsification"" appears to be adjusting the lower and upper boundaries of SUV or %iA/g scale bars to values which hide important aspects of the data. For example, a common way to achieve ""nice"" preclinical SPECT or PET images of tumor-bearing mice with apparently high contrast and low background is to set the lower limit &gt;0 in order to mask unwanted background. This is very commonly done for imaging results using radiolabeled antibodies, whose tumor uptake is high but the background is too. A typical sign of this mispractice is selecting ""low"" and ""high"" on scale bars, instead showing reasonable relative units (MBq/mL is also quite pointless because not comparable to other results). A second common way of avoiding to show the truth is presentation of slices only, instead of also showing MIPs that demonstrate overall biodistrobution. The main cause of the problem is that most publishers and reviewers do not strictly request the reporting of images with complete technical data, such as tracer type, injected activity, injected mass/molar amount, image type (slices vs. MIPs), scaling unit, upper and lower limits, etc.. Editors and reviewers apparently do not pay attention that the scaling is done in a non-distorting way (most importantly, with zero as lower limit, unless explicitly noted and a specific reason being given). Very often, imaging results are shown without any reasonable accompanying data. In order to improve this situation, editors must pay close attention to the problem, and also urge their reviewers to do so. Journals should set clear standards for image reporting, i.e., define the data that need to be reported with every image (see example list above)." |
| 12 | In complex cases, including ambiguous imaging findings may undermine the validity of your conclusions. Journals often have word count limitations that restrict the ability to fully discuss differential diagnoses, which can also pose ethical dilemmas. Even final pathology reports can be imperfect. Additionally, there is no established repository for submitting DICOM images or providing comprehensive clinical, laboratory, and pathology data that would enable reviewers to reach the same conclusions. Therefore, selectively presenting data seems to be a reasonable approach. |
| 13 | I think you may have a statistical bias in this survey, because I was selected despite not strictly doing research in a field that uses medical images. I believe you may have selected me because I co-authored a couple of articles on the use of CLINICAL medical imaging - this means that I analyzed datasets of diagnoses made with CT scans, but the analysis itself did not use images at all. This is why I had so many negative replies to the possible use of manipulation by myself or in the general field: I never analyse or use or publish medical images, I just used statistical datasets of data that have something to do with radiology or include variables that have to do with imaging - which is different. If you simply use the word "imaging" in article titles to select participants, you will get several similar participants and underestimate the use or danger of AI manipulation in the research fields that DO use images. Good luck anyway! |
| 14 | I think the main pressure for cherry picking results and enhancing images comes from industry collaborators or industry sponsored studies. These studies should be more highly scrutinized in journals and elsewhere. |
| 15 | I think that images alone cannot convey the reality, and that quantification of image features is a necessary step to approximate the result with precision. A requirement of quantification and statistical analysis of results would be helpful in working toward a remedy for this issue. |
| 16 | I suspect cherry picking is common, but that overt manipulation of images is relatively rare, but that there are many instances given the number of publications. I also suspect there is cherry picking of reported "positive" experiments that is an even larger problem. |
| 17 | I have never encountered any pressure by my institution. However, many images if they are not of high quality are not accepted. |
| 18 | I have known a colleague from a previous institution to withhold details about repeat subjects in an imaging study in order to be able to publish - this was &gt;5 years ago but I have never otherwise known of fabrication. I would love to see a presentation about this (with case studies and what to look out for!) at a conference or similar. Thanks. |
| 19 | I don't think image falsification is an isolated phenomenon. A large part of even the highest ranked journals are no longer impartial, but are bought/bribed to publish what the industry and others with sufficient funds will sacrifice to get their results and opinions out, regardless of whether the methods and results are valid, replicable and credible upon closer analysis. As a reviewer of multiple manuscripts and author of many articles, I see a gradual pervasive corruption in much of even the most respected medical literature. This is sad and a threat to medicine. We must hope that Europe, led by Northern Europe, will create its own impartial and objective counterpart to this - but unfortunately I see no signs of this yet. Perhaps this survey can be a first small step on the way? |
| 20 | I don't believe this is really a problem at this time, but I'm affraid it will become in the near future. I want to believe that it's possible to trust in the honesty of our colleagues, especially working in the medical field. Image falsification (as well as data falsification) is unacceptable and should be severly punished. |
| 21 | I do not think that AI-based tools to detect manipulated images would be reliable enough and may flag false positives. Similar to the issue of AI detection tools in writing. |
| 22 | I believe that enhancing contrast or brightness in a medical imaging is not a modification. On the other hand, to add some issues it is a modification and must be pursued |
| 23 | I am not sure that integrity checks are really possible given that the falsification might be in the description of treatment group or timepoint or something else that matters a lot but is not evident in the image per se. |
| 24 | I am involve in editorial boards of journals in the field of medical imaging. I've never heard of a paper with falsified images. I don't believe that this is because there are none. I think it's fairly rare but we are not looking for it. There needs to be a systematic way for journal to detect fake/falsified images. That way authors would think twice before submitting such images |
| 25 | Generally, I don't think medical images are manipulated as much as images in the basic science field. |
| 26 | Falsification of images is fraud, that is clear. |
| 27 | By the introduction of this questionairre I wasn't entirely sure about the subject you're researching. (in other words, what you mean with 'medical images', at first I thought o photographs and later animations but later I understood you alse mean graphs etc. Maybe clarify your introduction of this survey/guiding email. |
| 28 | As previous member of the committee for scientific integrity, i have witnessed several cases where image falsification was done (over the whole university only a handful), varying in severity degree. |
| 29 | Although manipulation of medical images in scientific publications should not be allowed, taking active measures to exclude or filter them out at the academic journal level seems necessary. |
| 30 | "Although I did not meet overt bad practices, I am simply not sure anymore about the integrity of the whole system, and probably became a little bit cynical: *Academic centers in general have a good research code, but the researcher has to follow the code. There are not many checks. * Scientific journals became business, but integrity in business is different from integrity in science. * In general the reviewing system is very poor. Reviewing can be considered to be a ""community service"". But serving the community is not very popular anymore (own career first). " |
| 31 | A comment to Q12: I think medical image falsification is rare in high impact publications but somewhat common in low impact journals. |
